# Supplementary material for: Evaluating the impact of open access policies on research institutions
Source: eLife. 2020 Sep 14;9:e57067. doi: 10.7554/eLife.57067 (PMC7536542; doi:10.7554/eLife.57067)
Supplement: Supplementary file 1. [file elife-57067-supp1.docx]

Supplementary file 1

# Technical infrastructure, reproducibility and provenance

Our technical infrastructure is constructed based on the aim to make openly available both the data and analysis code as much as possible. The data infrastructure is currently based in Google Cloud Platform, mainly utilising services in Google Storage, Google Functions, and Google Bigquery. Google Functions are used to extract data from the data sources’ APIs. The raw data is then stored on Google Storage for further processing. Google Bigquery is then used to merge and manipulate the raw data to process derived data in formats we require for further analysis.

Derived data used for analysis can be found at Zenodo (Huang et al., 2020b). Updated datasets will also be provided and will be found at the same location. Raw data is not provided to preserve the anonymity of institutions and respect the terms of service of data providers. The SQL queries and code used to generate the derived datasets are described below and available via Zenodo.

The main article, Supplementary Figures and this Supplementary Methodology were prepared as Jupyter notebooks to provide all analysis and visualisation code and maximise reproducibility. These notebooks are available at [Github](https://github.com/Curtin-Open-Knowledge-Initiative/institutional-oa-evaluation-2020) and [Zenodo](https://doi.org/10.5281/zenodo.3716063) (Huang et al., 2020c). All manipulation of derived data after import is explicitly conducted in the notebook. The notebook utilises a library for generating visualisations, which is provided with the notebooks. The only data manipulations performed by the visualisation library are to filter and re-shape the data for graphing.

Where possible we use a publicly available or defined data dump for our sources. In this article, data from Crossref, Unpaywall, GRID and Microsoft Academic were available as data dumps. The dumps used in this version (submitted version 19 March 2020) of these articles are as follows:

| Data Source | Dump Identity | Availability | License |
| --- | --- | --- | --- |
| Crossref | December 2019 | CR Metadata Plus Members <https://www.crossref.org/help/metadatasnapshots/> | cc0 |
| Unpaywall | 22 November 2019 | By request <http://unpaywall.org/products/snapshot> | cc0 |
| GRID | Release 2019-12-10 | <http://doi.org/10.6084/m9.figshare.11353022> | cc0 |
| Microsoft Academic Graph | 3 October 2019 | Via Azure <https://docs.microsoft.com/en-us/academic-services/graph/get-started-setup-provisioning> | ODC-By |

# Data sources

We integrate a variety of data sources in our data workflow to generate open access scores for a large set of universities. These sources include the following:

## Web of Science

Web of Science is a large pay-walled online scientific citation indexing service, maintained by Clarivate Analytics, with the coverage of more than 90 million records and 1.4 billion cited references. It is often harvested by universities to build-up their internal research information database. And, with much criticism, it is also used by various university rankings to evaluate performance (e.g., Academic Ranking of World Universities, CWTS Leiden Ranking, U-Multirank, etc). It stands as an important tool for various stakeholders of the academia. Web of Science includes a number of databases with varying levels of accessibility and information. For this study, we utilise the “organization-enhanced” search functionality to extract the list of publication metadata (hence, the corresponding DOIs) for each institution of interest (via our local access) from the Web of Science Core databases. Our access to Web of Science Core is restricted by our institutional subscription contract, which provides access to the following:

- Science Citation Index Expanded (SCI-EXPANDED) –1972-present
- Social Sciences Citation Index (SSCI) –1972-present
- Arts & Humanities Citation Index (A&HCI) –1975-present
- Conference Proceedings Citation Index- Science (CPCI-S) –1990-present
- Conference Proceedings Citation Index- Social Science & Humanities (CPCI-SSH) –1990present
- Book Citation Index– Science (BKCI-S) –2005-2012
- Book Citation Index– Social Sciences & Humanities (BKCI-SSH) –2005-2012
- Emerging Sources Citation Index (ESCI) –2015-present
- Current Chemical Reactions (CCR-EXPANDED) –1985-present
- (Includes Institut National de la Propriete Industrielle structure data back to 1840)
- Index Chemicus (IC) –1993-present

## Scopus

Scopus is an abstract and citation database launched by Elsevier in 2004. It provides subscription access and also produces a range of quality measures such as h-index, CiteScore and SCImago Journal Rank. For the purpose of our current work, we match each institution to its Scopus Affiliation ID and, subsequently, access the metadata of all publications related to each institution (again, via local access). The DOI, if existent, is extracted from each publication’s metadata.

## Microsoft Academic

Microsoft Academic, re-launched in 2016, is a replacement of the phased-out Microsoft Academic Search. It is a free public search engine for the academic literature and uses the semantic search technology developed by Microsoft Research. The database provides Affiliation Entity IDs for institutions. We utilise a snapshot of Microsoft Academic database to extract publication metadata related to each institution.

## Times Higher Education World University Rankings

This is an annual ranking produced by the Times Higher Education (THE) magazine. It is one of the most followed university rankings, together with the Academic Ranking of World Universities and Quacquarelli Symonds (QS) World University Rankings. The major components of the THE Ranking include its reputation survey and the citations data from Scopus. As a mean for comparisons, we selected the Top 1000 universities in the 2019 THE Ranking as our primary sample for calculating OA scores. This is supplemented with additional universities for countries with limited coverage in the primary sample. Subsets of universities are selected for longitudinal studies.

## Unpaywall

Unpaywall is a browser extension for finding free legal versions of paywalled research publications. It currently covers more than twenty-two million free scholarly articles and provides a large number of metadata related to OA, such as journal OA status (via DOAJ) and open license information. It has recently been integrated into the Web of Science and Scopus databases. For this study, each DOI of interest is matched with its metadata in Unpaywall for calculating the various OA status. Snapshots of the Unpaywall database are collected as part of data processing of this project.

## Crossref

Crossref is a not-for-profit official DOI registration agency of the International DOI Foundation. It is the largest (in terms of number of DOIs assigned) DOI registration agency in the world. It also provide JSON structured information surrounding each of its DOI, such as various related issue dates, and links between distributed content hosted at other sites. Our data collection process have resulted in several snapshots of the Crossref database. We primarily view Crossref DOIs as the basis for coverage of all global outputs in our study. We use the issued.date element in Crossref as the standardised indicator for publication year for each output in our data.

## Global Research Identifier Database (GRID)

This is an open access database of educational and research institutions worldwide. It assigns a unique GRID ID to each institution and, where applicable, to each level of the institutional hierarchy. The metadata includes information such as geo-coordinates, websites and name variants. These identifiers are adapted in our study to link the various bibliographic data sources and to unify the identification system.

# Description of data workflow and selection criteria

As discussed in the main article, our pragmatic approach is to include the widest coverage of outputs for each of the universities under consideration. This implies defining a *target population* for all potential research outputs, which is no trivial task. For this study, we choose to consider the set of all research outputs with Crossref DOIs as this target population. This is identified as the most practical approach that allows tracking and disambiguation of research objects using persistent identifiers. At the same time, it provides processes for both the standardisation of publication dates and the use of Unpaywall’s OA information.

We use universities listed in the top 1000 of the Times Higher Education World University Rankings as an initial sample for which to collect data. We then supplemented this with additional institutions, focussing on the United Kingdom and the United States. Finally we added additional universities in countries where our original sample had only one or two universities. For each of these countries we added a small number of additional universities, prioritising those with the largest number of research outputs recorded in Microsoft Academic.

Given that Microsoft Academic, Web of Science and Scopus have different internal institutional identifier systems, the next step is to map these identifiers. We first map each university to its unique ID in the Global Research Identifier Database (GRID). Subsequently, these universities’ internal identifiers for Microsoft Academic, Web of Science and Scopus are matched against the corresponding GRID IDs. This is trivial in the case of a Microsoft Academic database snapshot as each institution in its database is already matched against the corresponding GRID ID. For Web of Science and Scopus, manual website searches are required to retrieve Web of Science Organisation-Enhanced names and Scopus Affiliation IDs, respectively. ***Universities not identifiable in at least one of the three bibliographic data sources are not processed further***.

Queries were run via the respective APIs against Web of Science (via Organisation-Enhance name search) and Scopus (via Affiliation ID search) to extract metadata of all outputs affiliated to each university for the time frames 2000 to 2018. These are matched against outputs from a Microsoft Academic snapshot to result in a comprehensive set of outputs for each university. Subsequently, these are filtered down to include only objects with Crossref DOIs. This current set of universities is then further expanded to include additional universities from countries that had low representations in the initial sample, and goes through the same data collection process.

All collected Crossref DOIs are matched against an Unpaywall database snapshot for their open access information. This allows us to calculate total numbers for various modes of open access (e.g., number of Gold OA publications) for each university across different timeframes (using the “year” component of the Crossref “issued date” field). The Unpaywall information used to determine various open access modes is as displayed in Figure 1 of the main text. Crossref DOIs not found in Unpaywall are defaulted to be not open access and Crossref DOIs that do not have an “issued date” are removed from the process.

A comprehensive sensitivity analysis on the use of different sources for gathering research outputs, use of different Unpaywall versions, and the relations between confidence levels and sample size is provided in the companion white paper Huang et al. (2020b). There are changes based on which specific Unpaywall snapshot is used. This is partly due to real changes (e.g. release of works from repositories after embargo) and due to changes within the Unpaywall data system (examples include changes in upstream data sources such as journal inclusion or exclusion in the Directory of Open Access Journals (DOAJ), and internal changes such as improved repository calling or wider journal coverage). As this is a product of gradually improving systems underpinning Unpaywall we use the most recent available snapshot to provide the most up to date data in a reproducible and identifiable form in the main article.

To make comparable and fair analysis across universities, we have taken a necessarily subjective view on which universities to include in Figures 2 and 3 (along with their supplementary figures). We use the Šidák correction to control for the familywise error rate in multiple comparisons. The Šidák correction re-adjusts the size of the confidence intervals by increasing the confidence level to

100(1− *α*)^1/^*^m^*%

where *α* is the originally required level of confidence and *m* is the number of confidence intervals to be compared. In our case (*α* = 0*.*05 and *m* = 1), this essentially results in individual confidence intervals and margins of error being evaluated at 99.95%. Institutions with margin of error greater than 17, for any of total open access, gold open access or green open access, were also removed from data used to generate various figures. This is in addition to the conventional conditions for normal approximation (see Huang et al., 2020b).

## Overview of the data workflow and main tables

Within our Google Cloud Platform environment we maintain two sets of tables. The latest snapshot of each part of the dataflow is distinguished as _latest and specific snapshots are named for the date of production. We maintain a set of views within the Google Cloud BigQuery platform that are setup to query the latest available data. For this article we have created a specific snapshot, and that data is shared. Future versions of the shared dataset will provide updated snapshots.

The overall flow of data and the main tables and queries are described in Figures SM1 and SM2. The key table in our workflow is the institutions table. Figure SM1 details the main elements of the workflow that generates the institutions table. Figure SM2 details the downstream processing used to generate the derived datasets used in our analyses.

**Figure SF1: The generation of the institutions table.**


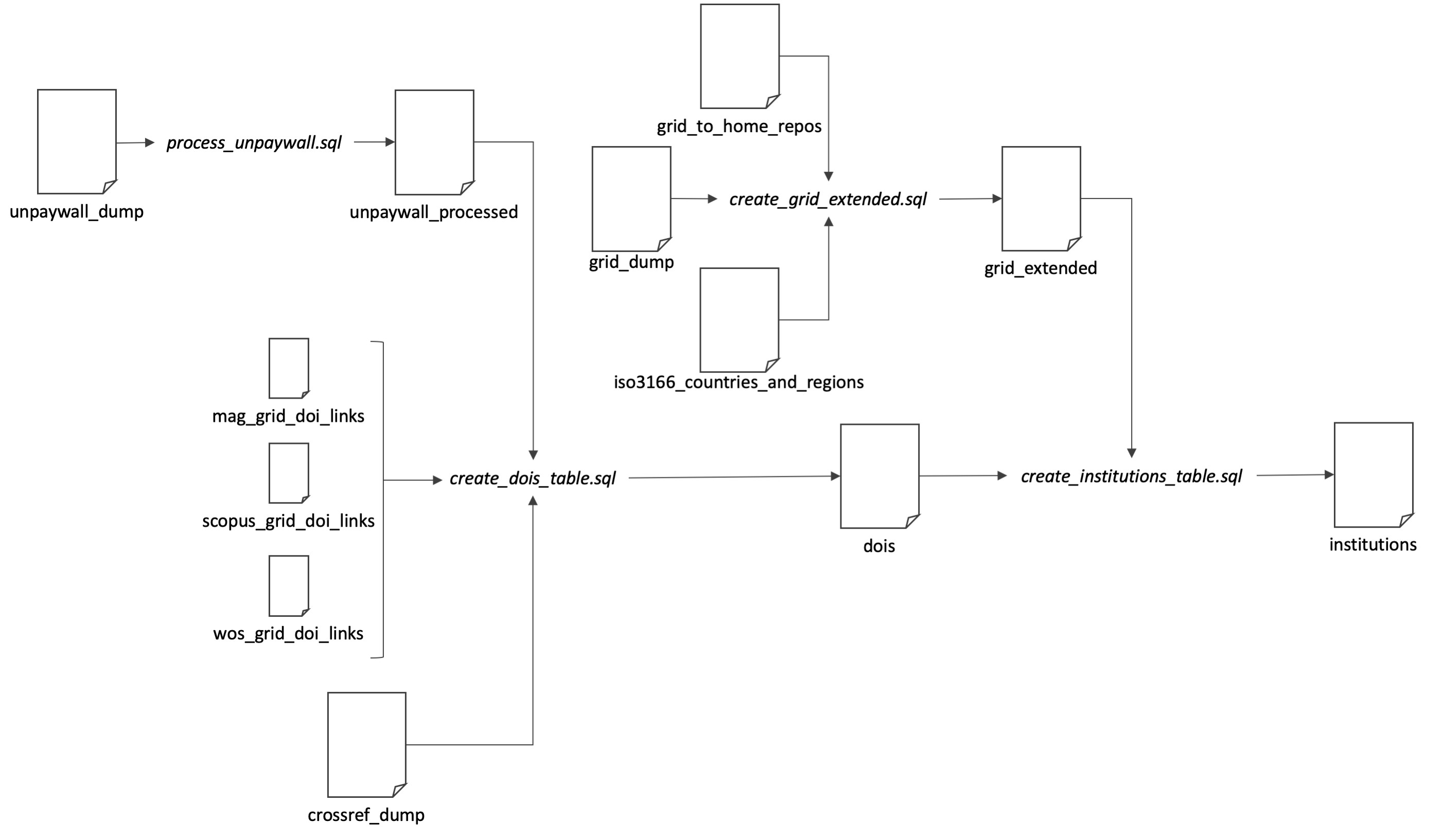


**Figure SF2: The generation of the derived datasets from the institutions table. Those datasets which are part of the publicly shared derived data are shown in green.**


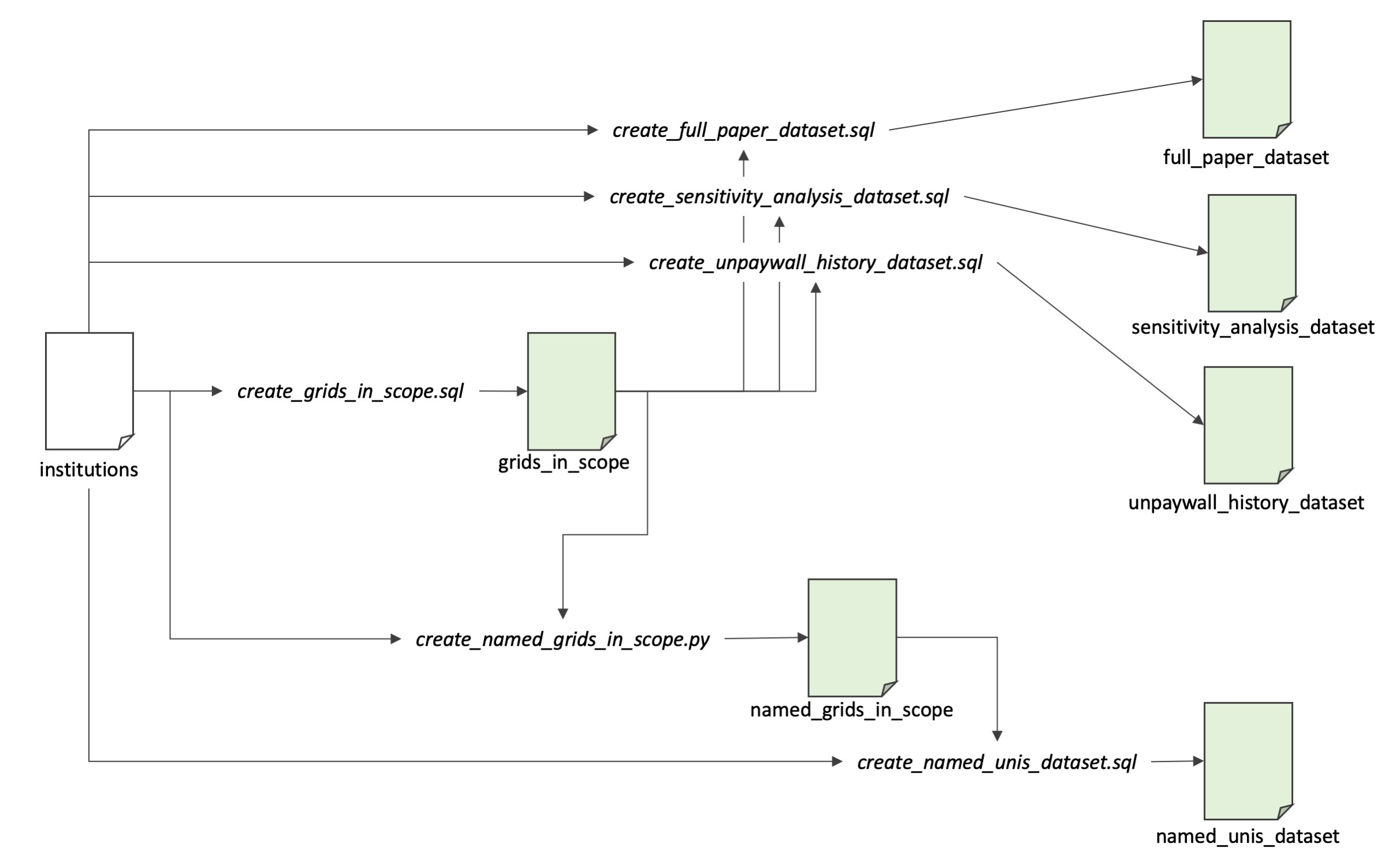


## Modes of open access

As summarised in the main article, we query each of three bibliographic data sources (Web of Science, Scopus and Microsoft Academic) for its list of research output affiliated to a given university, from years 2000 to 2018. Subsequently, this is filtered down to all objects with Crossref DOIs (by mapping against a Crossref data snapshot) and matched against Unpaywall metadata. We use the aggregated sets of DOIs for each year of publication (as per “issued date” in Crossref) to compute the counts for various OA modes using data from Unpaywall. The details of how different OA characteristics are calculated is shown in Figure 1 in the Results section of the main article. The details of the SQL query used to categorise OA status can be found below.

While there is a large body of literature on OA, the definitions of OA are quite diverse in detail. Policy makers and researchers may choose to use the OA terminology in different ways. Popular discrepancies include the coverage of journals without formal license of reuse and articles only accessible via academic social medias or illegal pirate sites. We use the following definitions for the modes of OA determined as part of our data workflow:

- **Total OA:** A research output that is free to read online, either via the publisher website or in an OA repository.
- **Gold:** A research output that is either published in a journal listed by the Directory of Open Access Journals (DOAJ), or (if journal not in DOAJ) is free to read via publisher with any license.
- **Gold DOAJ:** A research output that is published in a journal listed by DOAJ.
- **Hybrid:** A research output that is published in a journal not listed by DOAJ, but is free to read from publisher with any license.
- **Bronze:** A research output that is free to read online via publisher without a license.
- **Green:** A research output that is free to read online via an OA repository.
- **Green Only:** A research output that is free to read online via an OA repository, but is not available for free via the publisher.
- **Green in Home Repo:** A research output that is free to read online via the matched affiliation’s institutional repository.

It should be noted that these definitions are not always mutually exclusive in coverage. For example, an article can be both Gold OA and Green OA. On the other hand, the set of all Gold OA and the set of all Green Only OA do not have any common element by definition. In the main text of this article, we only report the categories: Total OA, Gold, Hybrid, Green, Green in Home Repo. A sensitivity analysis of the use of alternative categories of OA can be found in the companion white paper.

The full query that processes the Unpaywall data to processed open access status is as follows and can also be found in the Data and Queries package at Zenodo (Huang et al., 2020c).

**SELECT**

doi,

year,

genre **as** output_type,

publisher,

journal_name,

is_oa,

journal_is_in_doaj **as** is_in_doaj,

**IF**(is_oa, **IF**(best_oa_location.license **IS** **NOT** NULL, TRUE, FALSE), FALSE)

**as** has_license,

**IF**(is_oa, **IF**(best_oa_location.license **IS** **NOT** NULL,

**IF**( STARTS_WITH(best_oa_location.license, "cc"), TRUE, FALSE), FALSE),

FALSE)

**as** is_cclicensed,

best_oa_location.license **as** has_specified_license,

**IF**(journal_is_in_doaj, TRUE, FALSE) **as** gold_just_doaj,

**IF**(journal_is_in_doaj **OR**

(best_oa_location.host_type **=** "publisher" **AND**

best_oa_location.license **is** **not** null **AND** **not** journal_is_in_doaj), TRUE, FALSE)

**as** gold,

**IF**(**not** journal_is_in_doaj **AND**

best_oa_location.host_type **=** "publisher" **AND**

best_oa_location.license **is** **not** null, TRUE, FALSE)

**as** hybrid,

**IF**((**SELECT** **COUNT**(1) **FROM** UNNEST(oa_locations) **AS**

location **WHERE** location.host_type **IN** ('repository')) **>** 0, TRUE, FALSE)

**as** green,

(**SELECT** **COUNT**(1) **FROM** UNNEST(oa_locations) **AS** location **WHERE** location.host_type **IN**

('repository')) **>** 0 **AND**

(**SELECT** **COUNT**(1) **FROM** UNNEST(oa_locations) **AS** location **WHERE** location.host_type **IN**

('publisher')) **=** 0

**as** green_only,

(**SELECT** **COUNT**(1) **FROM** UNNEST(oa_locations) **AS** location **WHERE** location.host_type **IN**

('repository')) **>** 0 **AND**

**NOT** (**NOT** journal_is_in_doaj **AND**

(best_oa_location.host_type **=** "publisher" **AND** best_oa_location.license **is** **not** null))

**as** green_only_ingnoring_bronze,

**IF**(is_oa, **if**(best_oa_location.host_type **=** "publisher" **AND**

best_oa_location.license **is** null **AND**

**not** journal_is_in_doaj, TRUE, FALSE), FALSE)

**as** bronze,

ARRAY((**SELECT** url **FROM** UNNEST(oa_locations) **WHERE** host_type **=** "repository"))

**as** repository_locations,

best_oa_location.url_for_landing_page,

best_oa_location.url_for_pdf

**FROM**

`academic-observatory-telescope.unpaywall.unpaywall_2018_09_24`

## Identification of grids in scope

Our full dataset includes all those institutions for which a GRID is recorded in Microsoft Academic Graph. In this article we have focussed on a set of institutions seeded from the top 1000 institutions in the THE World University Ranking supplemented for greater geographical coverage and deeper coverage of specific countries. We identify those GRIDs in scope for this article by identifying GRIDs for which we have additionally collected data from Scopus and Web of Science. As there are a small number of non-university research institutions in this set we explicitly exclude them.

The query that generates the grids_in_scope table is as follows:

**SELECT**

id, **MAX**(name) **as** name, **MAX**(total_years) **as** **count**

**FROM** `academic-observatory.institution.institutions_2020_02_12`, UNNEST(years) **as** years

**WHERE**

years.just_scopus.total **>** 0 **AND** years.just_wos.total **>** 0 **and**

id **not** **in** ('grid.7327.1', *# Council for Scientific and Industrial Research*

'grid.415021.3', *# South African Medical Research Council*

'grid.417715.1', *# Human Sciences Research Council*

'grid.415861.f', *# Uganda Virus Research Institute*

'grid.428711.9', *# Agricultural Research Council of South Africa*

'grid.452736.1') *# South African National Biodiversity Institute*

**GROUP** **BY**

id

**ORDER** **BY** **count** **ASC**

## Identification of named universities in the top 100s

The dataset containing named universities are all of those that fall, for any year from 2013-2018 inclusive, into the top 100 of:

1. Overall percentage of OA (i.e., Total OA)
2. Percentage of Green OA
3. Percentage of Gold OA

This group is identified using the following query and code, which is used to generate the table ‘named_institutions_in_scope’ which is also provided in the shared dataset. We select 110 from each category due to the filtering downstream of smaller institutions.

This table is generated by a small python script as follows:

**import** pandas **as** pd

**import** pandas_gbq

template **=** """

SELECT id, name

FROM `academic-observatory.institution.institutions_2020_02_12`, UNNEST(years) as years

WHERE

years.published_year = {} AND

id in (SELECT id FROM `open-knowledge-publications.institutional_oa_evaluation_2020.

grids_in_scope_2020_02_12`)

ORDER BY (SAFE_DIVIDE({}, combined.total)) DESC LIMIT 110

"""

df **=** pd.DataFrame()

**for** year **in** range(2013,2019):

**for** oa_type **in** ['combined.oa', 'combined.green', 'combined.gold']:

sql **=** template.format(year, oa_type)

result **=** pandas_gbq.read_gbq(sql, project_id**=**'open-knowledge-staging')

df **=** df.append(result, ignore_index**=True**)

unique **=** df.drop_duplicates()

unique.to_gbq('institutional_oa_evaluation_2020.named_grids_in_scope_2020_02_12',

project_id**=**'open-knowledge-publications')

## Generation of the derived datasets

The four main datasets that are publicly shared are generated directly from the institutions table using either the grids_in_scope or the named_grids_in_scope tables to provide a filter for the set of institutions. The queries have some minor differences to provide the data of interest in each case. All four queries are provided in the Data and Queries package available at Zenodo. Here we show the query for the generation of the full_paper_dataset. We use a salt to generate the anonymised IDs for each university.

**SELECT**

*-- Replace ###salt### with your own salt. This is used to anonymise institutions*

*-- in our article.*

TO_HEX(MD5(CONCAT(id, '', '###salt###'))) **as** id,

country,

country_code,

region,

subregion,

published_year,

combined.total **as** total,

combined.oa **as** total_oa,

combined.green **as** green,

combined.gold **as** gold,

combined.hybrid **as** hybrid,

combined.bronze **as** bronze,

combined.green_only **as** green_only,

combined.green_in_home_repo **as** green_in_home_repo,

SAFE_DIVIDE(combined.oa, combined.total) ***** 100 **as** percent_oa,

SAFE_DIVIDE(combined.green, combined.total) ***** 100 **as** percent_green,

SAFE_DIVIDE(combined.gold, combined.total) ***** 100 **as** percent_gold,

SAFE_DIVIDE(combined.hybrid, combined.total) ***** 100 **as** percent_hybrid,

SAFE_DIVIDE(combined.bronze, combined.total) ***** 100 **as** percent_bronze,

SAFE_DIVIDE(combined.green_only, combined.total) ***** 100 **as** percent_green_only,

SAFE_DIVIDE(combined.green_in_home_repo, combined.total) ***** 100 **as** percent_in_home_repo,

**FROM**

`academic-observatory.institution.institutions_2020_02_12` **as** institutions,

UNNEST(years) **as** years

**WHERE**

years.published_year **>** 2000 **and**

years.published_year **<** 2020 **and**

institutions.id **in** (**SELECT** id **FROM** `open**-**knowledge**-**publications.institutional_oa_

evaluation_2020.grids_in_scope_2020_02_12`)
